# Psychosocial Impact of the COVID-19 Pandemic on Healthcare Workers and Initial Areas of Action for Intervention and Prevention—The egePan/VOICE Study

**DOI:** 10.3390/ijerph181910531

**Published:** 2021-10-07

**Authors:** Lucia Jerg-Bretzke, Maximilian Kempf, Marc Nicolas Jarczok, Katja Weimer, Christian Hirning, Harald Gündel, Yesim Erim, Eva Morawa, Franziska Geiser, Nina Hiebel, Kerstin Weidner, Christian Albus, Petra Beschoner

**Affiliations:** 1 Department of Psychosomatic Medicine and Psychotherapy, Ulm University Medical Center, 89081 Ulm, Germany; maximilian-1.kempf@uni-ulm.de (M.K.); marc.jarczok@uni-ulm.de (M.N.J.); katja.weimer@uni-ulm.de (K.W.); christian.hirning@uni-ulm.de (C.H.); harald.guendel@uniklinik-ulm.de (H.G.); petra.beschoner@uniklinik-ulm.de (P.B.); 2 Department of Psychosomatic Medicine and Psychotherapy, University Hospital Erlangen, Friedrich-Alexander University Erlangen-Nürnberg (FAU), 91054 Erlangen, Germany; Yesim.Erim@uk-erlangen.de (Y.E.); eva.morawa@uk-erlangen.de (E.M.); 3 Clinic and Polyclinic for Psychosomatic Medicine and Psychotherapy, University Hospital Bonn, 53127 Bonn, Germany; franziska.geiser@ukbonn.de (F.G.); Nina.Hiebel@ukbonn.de (N.H.); 4 Department of Psychotherapy and Psychosomatic Medicine, Faculty of Medicine, Technische Universität Dresden, 01307 Dresden, Germany; Kerstin.Weidner@uniklinikum-dresden.de; 5 Department of Psychosomatics and Psychotherapy, Medical Faculty and University Hospital, 50924 Cologne, Germany; Christian.albus@uk-koeln.de

**Keywords:** COVID-19, occupational stress, healthcare workers, mental health

## Abstract

Introduction: Epidemics lead to an increase in occupational stress and psychological strain among healthcare workers. However, the impact of a pandemic outbreak on healthcare systems is yet to be clearly defined. Therefore, this work aims to describe and analyze specific areas of workload among different groups of healthcare workers during the first wave of the COVID-19 pandemic. Methods: A sample of N = 8088 persons working in the German-speaking healthcare sector participated in the VOICE/egePan online survey, which addressed the impact of the COVID-19 pandemic during the second quarter of 2020. We used 15 self-constructed items, based on the work of Matsuishi et al. (2012), to identify potential COVID-19-specific topics. Results: N = 7542 records of healthcare workers were analyzed. Of these, 60.80% reported, retrospectively, an increase in stress since the outbreak of the pandemic. Problem areas tended to be indicated more frequently by the women surveyed than by the men. Nurses, paramedics and medical technicians reported the highest fear of infecting others while physicians reported the highest fear of physical or mental exhaustion. With respect to age, older respondents indicated less fear and felt more protected. Men and people living alone were more likely to use dysfunctional coping strategies. Migrants reported a higher fear of becoming infected or infecting others as well as they reported about increased levels of smoking. Discussion: Retrospectively, the COVID-19 pandemic led to an increase in stress among healthcare workers. Problem areas have different focuses with regard to different living situations, environmental conditions and professions. In order to lay the best basis for healthy and efficient work, it seems necessary to take measures especially tailored to the needs of different groups of healthcare workers.

## 1. Introduction

A large number of studies show that epidemics are associated with a high level of mental stress among healthcare workers [1,2,3]. In the current COVID-19 pandemic, international studies also show high levels of stress and psychological symptoms among healthcare workers [2,4,5]. A review by Spoorthy et al. shows that the majority of studies identified symptoms of stress, anxiety and depression in association with the COVID-19 pandemic, and concludes that the pandemic can be seen as an independent stress factor among healthcare workers [6]. Since the beginning of the pandemic, several authors have already emphasized its large impact on the mental health of healthcare workers. Healthcare workers are in the spotlight in the fight against the pandemic and the treatment of patients with a life-threatening illness, for which there are only limited treatment options. They are confronted with an increased workload, a change in tasks and thus considerable occupational stress. At the same time, they can often do little with limited equipment. They are exhausted, but are still confronted with dying, death and immense suffering in substantial amounts. Some have to make decisions in triage situations that push them, morally, to their limits. In addition, there is the risk of infection and the fear of infecting relatives, e.g., in March 2020 it had already been reported that over 30,000 Chinese physicians have been infected with SARS-CoV-2, of which more than 30 died from the infection, especially when adequate protective equipment is not available [7,8,9]. 

In addition, the fear of infecting oneself can develop into a form of health anxiety: the excessive fear of getting or having the disease leads to symptoms such as coughing and breathing difficulties. In the current pandemic, there is now a real possibility that these could be incipient COVID-19 symptoms. Therefore, the fear cannot yet be considered pathological, yet it can be very mentally straining [10].

A growing number of international studies shows that these stressful working conditions and the resulting mental strain caused by the pandemic leave traces on employees: A cross-sectional web-based survey of over 2000 health workers in China showed a high prevalence of mental health problems. The prevalence of general mental health problems was 57%, anxiety 46% and depression 44%. Compared to health workers who did not work on the frontline, those on the frontline had a higher risk of anxiety and mental health problems overall [11]. Additionally, an Italian study on over 600 healthcare workers showed that healthcare workers who work in direct contact with COVID-19 patients suffer more from stress, burnout, anxiety and depression [12]. A Spanish study confirmed this for the burnout risk factor and identified young, female doctors as a particularly vulnerable group [13]. Further studies on healthcare workers in Spain showed high rates of mental distress in terms of burnout symptoms among physicians in hospitals (39%) and employees in nursing homes (up to 54%) in the COVID-19 pandemic. More than 90% of physicians surveyed wished for psychological support in their workplace [14,15].

Individual studies in Germany describe the pandemic’s impact on mental health and demonstrate links between the pandemic and various clinical symptoms. In their study Skoda et al. compared individuals in healthcare to those outside of it and found that individuals outside of healthcare were less impacted in terms of depressive symptoms and anxiety. Within the group of healthcare workers, nurses showed the highest level of mental stress [16]. The results of a study on physicians and nurses at a university hospital in Germany also point in this direction. The authors state that nurses were more mentally stressed than physicians and that, in particular, the increased workload and prolonged contact with COVID-19-infected patients were significant influencing factors [17].

Other publications confirm the links between the pandemic and mental stress among healthcare workers (depressiveness, PTSD symptoms) [18,19]. A Turkish survey of physicians conducted during the first wave associated different conditions with elevated depression and anxiety scales. These conditions included the female gender, low work experience, increase in work hours, increase in number of COVID-19 patients and low support from colleagues and supervisors [20]. A cross-sectional study on healthcare workers in China also yielded high prevalence rates for depression and anxiety (46.04 and 44.37, respectively) in the current pandemic [11]. Whether these factors can be applied to the German healthcare system and professions other than physicians is questionable. 

Both international and German studies found an increased burden on healthcare workers due to the COVID-19 pandemic as well as limitations in the quality of care for those affected [21,22].

Furthermore, international studies emphasize the need for intervention options [23,24] and also draw conclusions based on previous pandemics [14,15,25]. However, to our knowledge, no empirical study has been conducted so far which addresses the specific stress factors of the COVID-19 pandemic among healthcare workers independent of clinical symptoms and recommends empirically supported options for supporting those affected.

Stress and its negative impact on the psychological health of medical personnel as well as medical malpractice in hospitals affect the quality of patient care and patient satisfaction [26,27,28,29]. Consequently, protecting the psychological health of healthcare workers appears essential to the proper management of a pandemic situation. In order to provide measures to meet the different needs and conditions of healthcare workers, specific stress factors must be determined.

Thus, our work aimed to identify the psychosocial impact of the COVID-19 pandemic, independent of clinical stage, among different groups of healthcare workers. It is assumed that psychological stress increases as a result of the pandemic. We relied on data from previous pandemics to identify the relevant problem areas and protective factors, and examined which of these factors are prominent among German healthcare workers. It is assumed that the problem areas have different focuses and characteristics depending on the profession. It is speculated that stress factors specific to the COVID-19 pandemic are treated differently depending on the living situation of the respondent and environmental conditions. The results will be used to identify initial areas of action in Germany based on empirical data.

## 2. Materials and Methods

The present study results are part of the VOICE online study, which was conducted as part of the NUM (Network of University Medicine)–egePan research. 

From mid-April to 5th of July 2020 a total of N = 8088 employees from the German-speaking healthcare sector were surveyed for the first time. Data records that did not include relevant information (age, gender) or responses to the items of the standardized instruments were removed entirely.

Data were collected online and anonymously using the Unipark platform. The participants agreed to participate in the study by completing the questionnaires. The online questionnaire took around 20 min to complete. Approvals have been obtained from the ethics committees of the University of Ulm and cooperating hospitals in Erlangen, Bonn, Cologne and Dresden.

The questionnaire contained demographic questions such as age and gender, questions on personal and professional living situations and workloads (see Table 1) as well as items on specific problems and protective factors in the COVID-19 pandemic (see also Table 2 and Table 3). We developed these items based on the work of Matsuishi et al. (2012), who investigated the psychological impact of the 2009 H1N1 epidemic on healthcare workers [30]. They asked the participants 16 stress-related questions and conducted a factor analysis on their responses. The Cronbach’s alpha coefficient of the 16 stress-related questions was α = 0.84 (N = 1625), indicating good internal consistency and acceptable reliability. Due to the lack of sophisticated instruments to measure pandemic-specific stress and protective factors, we used the results to formulate 15 items for our survey in the most evidence-based manner possible with a 5-point Likert scale from 1 = disagree fully to 5 = fully agree. Thirteen items assess COVID-19-related problems and two items assess protective factors during the pandemic (see Table 2 and Table 3). In our sample, the Cronbach’s alpha of the 13 problem-related questions of α = 0.78 indicates a comparable and reasonable internal consistency and acceptable reliability.

General stress before and during the pandemic was determined by the questions “How much stress did you feel before the COVID-19 pandemic began?” and “How much stress have you felt due to the COVID-19 pandemic over the past 2 weeks, including today?” with a 5-point Likert scale from 1 = not at all to 5 = very strong.

Data were processed and analyzed using IBM SPSS Statistics for Windows Version 27.0 (IBM Corp., Armonk, NY, USA) and Stata SE 15.1 (StateCorp LLC, College Station, TX, USA). Significance level was set to α = 0.05. Normal distribution of variables was assessed with Kolmogorov–Smirnoff tests and visual inspection of normal quantile–quantile plots. We introduce our results with the distribution of respondents of the different occupational groups prior to relevant characteristics, followed by descriptive analyses for relevant outcome variables (self-rated stress levels, specific surveyed problems and protective factors associated with COVID-19). Following these analyses, differences between the groups of interest are examined. We start with the comparison of the gender groups, for which *t*-tests were used to show relevant differences in the outcome variables mentioned above. In a further step, differences in the occupational groups are first presented by means of descriptive statistics and illustratively by means of a semantic differential. In addition, differences with regard to the problems and protective factors associated with COVID-19 will be shown by calculating ANOVAs. In order to control for multiple testing, p-values were adjusted for the false discovery rate according to Benjamini and Hochberg [31] and reported in Table 4. Because of the importance of contact with a virus in a pandemic, differences between individuals with and without contact with regard to the problems and protective factors associated with COVID-19 are presented using *t*-tests. Finally, other characteristics of the participants that may carry additional burden due to the changes within the pandemic (e.g., migration background, home office, care of dependents or children as well as age) are used and differences in the pre-defined relevant problem areas and protective factors related to COVID-19 are illustrated using mean differences and *t*-tests. 

## 3. Results

### 3.1. Composition and Description of the Sample 

A total of N = 8088 employees participated. Of these, a total of N = 7542 individuals also answered questions related to COVID-19 and additional stress-related questions. Demographic data on the various occupational groups can be found in Table 1.

### 3.2. Stress Factors and Problems Due to COVID-19 and Protective Factors

While 17.10% (N = 1380) reported less stress and 22.10% (N = 1780) reported no change in stress since the beginning of the pandemic, 60.80% reported more stress.

The greatest sources of stress from the COVID-19 pandemic included fear of infecting loved ones and family followed by physical or mental exhaustion and change in tasks. This is shown in Table 2, in which the mean values and standard deviations of the participants’ answers to COVID-19-specific items (with options to answer from 1 = strongly disagree to 5 = strongly agree) are presented.

Table 3 shows the sample distribution (reported as *n* (%)) of COVID-19-associated problems and protective factors across the various response options.

### 3.3. Gender Differences

Based on the descriptive comparisons it is evident that women (N = 5747, 76.38%) in the pandemic show similar or more pronounced levels of stress than men (N = 1777, 23.62%) in almost all problem areas. Men and women agreed only in their assessment of patient safety (item 12, M_m/f_ = 2.10) and use of antidepressants/sedatives (item 15, M_m_ = 1.13/M_f_ = 1.14). With regard to the consumption of alcohol and nicotine (items 13 and 14), men tended to report more consumption (M_13_ = 1.46, SD_13_ = 1.10; M_14_ = 1.73, SD_14_ = 1.16) than women (M_13_ = 1.38, SD_13_ = 1.00; M_14_ = 1.59, SD_14_ = 1.07). Men reported feeling slightly more protected by their employer (M_6_ = 3.30, SD_6_ = 1.22) than women (M_6_ = 3.24, SD_6_ = 1.16), while there was no difference in how protection by national/local authorities was perceived (item 5). Cited gender differences were significant at a level of at least *p* < 0.05. It is worth mentioning that the effect size (Cohen’s d), varying between d = |0.004| (item 5) to d = |0.230| (item 9), was on a negligible to small level.

### 3.4. Comparison of Different Occupational Groups 

**Figure 1:**
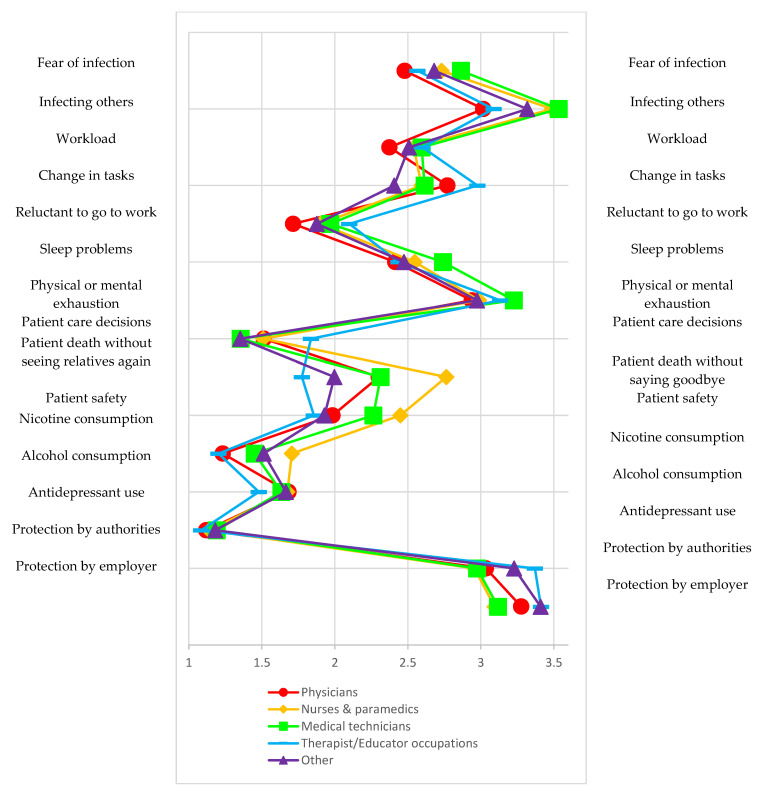
Distribution of characteristics of COVID-19-associated problems and protective factors among different occupational groups.

The comparison of occupational groups shows that therapeutic and educational professions (N = 1601, 21.2%) were stressed the most by changes in professional tasks (item 4) (M = 2.98, SD = 1.32). The fear of infecting themselves or others was lowest (M_1_ = 2.48, SD_1_ = 1.22; M_2_ = 3.02, SD_2_ =1.34) among physicians (N = 1906, 25.3%) and highest among nurses and paramedics (M_1_ = 2.73, SD_1_ = 1.30; M_2_ = 3.49, SD_2_ =1.34) as well as medical technicians (M_1_ = 2.86, SD_1_ = 1.24, M_2_ = 3.53, SD_2_ =1.30). Physicians (M_7_ = 1.72, SD_7_ = 1.02) were the least likely to feel reluctant about going to work. Medical technicians (N = 1727, 22.9%) were the most likely to report mental or physical exhaustion (M_9_ = 3.23, SD_9_ = 1.27). Compared to other professions, nurses and paramedics (N = 1422, 18.9%) experienced the most stress caused with the thought that patients might die without seeing relatives again (M_11_ = 2.76, SD_11_ = 1.45), while therapeutic and educational professions experienced the least (M_11_ = 1.78, SD_11_ = 1.24). In addition, nurses and paramedics (M_13_ = 1.71, SD_13_ = 1.29) reported the greatest increase in nicotine use, while antidepressant use was largely the same among the different professions (M_15_ = (1.08; 1.19)), and only the therapeutic and educational professions reported a lower/slight increase in alcohol use (M_14_ = 1.48, SD_14_ = 0.94). The differences in the various problem areas and protective factors are shown in Figure 1. The specified differences between the groups were statistically significant across all items (see Table 4). 

### 3.5. Comparison of Persons with Contact versus Those without Contact

When considering individuals who came into contact with infected individuals or contaminated materials during the first wave (N = 3601, 47.7%), it becomes apparent that such contact leads all those surveyed to report an increase in COVID-19-associated problem areas and a decrease in perceived protection. Excluded from this were responses to questions on the reluctance to go to work (M_Contact_ = 1.92, SD_Contact_ = 1.11 vs. M_No contact_ = 1.90, SD_No contact_ = 1.10) or the fear of having to decide who receives care and who does not (M_Contact_ = 1.53, SD_Contact_ = 0.97 vs. M_No contact_ = 1.53, SD_No contact_ = 0.99). Mean differences between the contact versus no-contact groups (N = 3941, 52.3%) were significant at least at a level of *p* < 0.05. Effect sizes varied between d = |0.056| and d = |0.348|.

### 3.6. Other Characteristics (the Differences Depicted Refer to the Reference Group in Each Case, e.g., Respondents with a Migrant Background versus Those without One)—Home Office, Migrant Background, Living Alone, Caregiving Responsibilities and Age

Respondents with a migrant background were more likely to report fear of becoming infected (M = 2.80, SD = 1.31) than respondents without a migrant background (M = 2.64, SD = 1.23, *p* < 0.001, d = 0.13). 

People unable to work from home and people with a migrant background were more afraid of infecting others (M = 3.32, SD = 1.34 or M = 3.41, SD = 1.41) than respondents who were able to work from home (M = 3.12, SD = 1.34, *p* < 0.01, d = 0.15) or do not have a migrant background (M = 3.26, SD = 1.34, *p* < 0.01, d = 0.112). With respect to age, older respondents indicated less fear of infecting others (see Table 5).

Respondents with caregiving responsibilities suffered more from the increase in workload (M = 2.55, SD = 1.62) than those without (M = 2.46, SD = 1.21, *p* < 0.01, d = 0.075). 

Respondents with caregiving responsibilities and respondents able to work from home reported suffering more from the change in tasks (M = 2.76, SD = 1.33 or M = 2.84, SD = 1.29) than respondents without caregiving responsibilities (M = 2.61, SD = 1.28, *p* < 0.01, d = 0.12) and those unable to work from home (M = 2.67, SD = 1.32, *p* < 0.01, d = 0.13). 

The younger the age, the more respondents suffered from the thought that patients could die without seeing their relatives again and felt that patient safety suffered from the higher workload.

The groups with a migrant background, without caregiving responsibilities or living alone each reported smoking more than before (M = 1.55, SD = 1.19; M = 1.47, SD = 1.11; or M = 1.59, SD = 1.21) compared to those without a migrant background (M = 1.38, SD = 1.00, *p* < 0.01, d = 0.17), with caregiving responsibilities (M = 1.35, SD = 0.96, *p* < 0.01, d = 0.12) or not living alone (M = 1.35, SD = 0.96, *p* < 0.01, d = 0.24). 

While increased alcohol consumption decreased with age, it increased more for those living alone (M = 1.70, SD = 1.16) than for those not living alone (M = 1.61, SD = 1.08, *p* < 0.01, d = 0.083). Furthermore, respondents living alone reported using more antidepressants/sedatives (M = 1.19, SD = 0.68) than those not living alone (M = 1.12, SD = 0.56, *p* < 0.01, d = 0.12).

With regard to protective factors, it was found that protection by authorities and employers was rated more strongly with increasing age. In contrast, respondents unable to work from home (M_Protection by authorities_ = 3.06, SD_Protection by authorities_ = 1.08; M_Protection by employer_ = 3.19, SD_Protection by employer_ = 1.17), with a migrant background (M_Protection by authorities_ = 2.96, SD_Protection by authorities_ = 1.07; M_Protection by employer_ = 3.11, SD_Protection by employer_ = 1.19) or living alone (M_Protection by authorities_ = 3.05, SD_Protection by authorities_ = 1.08; M_Protection by employer_ = 3.14, SD_Protection by employer_ = 1.15) were less likely to say they felt protected by the actions of national and local authorities or their employer than respondents who were able to work from home (M_Protection by authorities_ = 3.26, SD_Protection by authorities_ = 1.02, *p* < 0.01, d = 0.19; M_Protection by employer_ = 3.46, SD_Protection by employer_ = 1.16, *p* < 0.01, d = 0.23), without an immigrant background (M_Protection by authorities_ = 3.12, SD_Protection by authorities_ = 1.07, *p* < 0.01, d = 0.15; M_Protection by employer_ = 3.26, SD_Protection by employer_ = 1.18, *p* < 0.01, d = 0.13) or not living alone (M_Protection by authorities_ = 3.12, SD_Protection by authorities_ = 1.07, *p* = 0.02, d = 0.07; M_Protection by employer_ = 3.28, SD_Protection by employer_ = 1.19, *p* < 0.01, d = 0.12). In addition, respondents with caregiving responsibilities reported (M = 3.30, SD = 1.19) feeling more protected by their employer than respondents who did not care for children or dependents (M = 3.17, SD = 1.14, *p* < 0.01, d = 0.10). 

## 4. Discussion

The present survey investigated the impact and potential effects the first wave of the COVID-19 pandemic had on healthcare workers in Germany between mid-April and the 5th of July 2020. For the majority of respondents (60.8%) the pandemic led to a significant increase in stress. This result points in the same direction as the data from international studies on the mental stress of healthcare workers [25,32,33,34]. Meanwhile there is also a growing awareness of the need for action in terms of individual and institutional support measures [35,36,37]. 

The items on COVID-19-associated problems and protective factors in our study, which were formulated in advance, found varying degrees of agreement among the respondents at the time the survey was conducted. The most pronounced problems overall were the fear of infecting relatives, stress due to changes in tasks, physical or mental exhaustion and, as a protective factor, perceived protection by employers. Similar findings were reported by Belanti et al., 2020, who found that workload and lack of emotional support at the workplace were particularly predictive of mental distress in terms of burnout among nurses during the pandemic [38]. A study on French HCWs in intensive care identified the fear of infecting themselves or relatives with the virus as a predictor of various mental health problems [39]. Additionally, in their systematic review, Skolaridis et al. postulate that the fear of infecting relatives is an important stress factor for healthcare workers in epidemics and pandemics. To offset this factor they describe opportunities for employers to isolate themselves as a measure, as well as psychological support offers for individual stress prevention [40].

If the sample is considered for specific characteristics, it becomes apparent that problem areas involving stress and anxiety tended to be indicated more frequently by the women surveyed than by the men, as also shown in the study by Torrente et al. and Yang et al. [13]. The systematic review by Pappa et al. also found a higher prevalence rate of anxiety and depression in women in the COVID-19 pandemic [41]. Although this may be due to the tendency of women to identify sources of stress more clearly [42,43], it should be noted that the survey included significantly more women and may be biased as a result. In contrast, the men surveyed were more likely to report an increase in alcohol or nicotine use. This may indicate that men are more likely to use dysfunctional coping strategies, which is also reflected in the prevalence figures regarding the use of alcohol and nicotine [44]. The highest increase in nicotine use and the most stress during the pandemic were reported by nurses/paramedics when faced with choosing who would receive treatment and who would not. In general, substance use, especially alcohol consumption, appears to have increased in the pandemic [45]. A link between the increase in use of smoking and the greater expression of anxiety symptoms is shown by study results from Turkey by Kabasakal [46]. Based on an online survey, Vanderbruggen et al. found that the consumption of alcohol, and especially smoking, increased significantly in the general population during the pandemic [47]. 

Male respondents also tended to feel more protected by the measures taken by their employers. This may be explained by the imbalance that still exists between men and women in the workplace environment. For instance, men earn more than women [48] and this imbalance could be reflected in the extent to which the protective measures of employers are trusted. A major burden for health workers in pandemics is the fear of becoming infected themselves [30]. When the different occupational groups surveyed are compared, it is clear that physicians reported the least fear of infecting themselves or others while nurses/paramedics and medical technicians reported the most, which can be intuitively explained by the latter having closer or more intense contact with infected patients and contaminated materials. This observation fits with the findings of Zhang et al., who found a higher prevalence of mental stress symptoms, such as anxiety and depression, in healthcare workers with direct contact to COVID-19-infected patients (N = 927) compared with nonmedical healthcare workers (N = 1255) [49]. A study on 310 Canadian HCWs also showed that the prevalence of psychosocial stress was highest among registered nurses (75.7%) and lowest among physicians (49.4%) [50]. Additionally, a systematic review by Busch et al. also found that frontline staff were primarily concerned about infecting themselves (47%) or passing the virus to family members (60%) [34]. Early and quick intervention by employers is needed to prevent a possible development as described by Tyrer: COVID-19 anxiety, which in turn is a prolonged mental strain [10].

Even respondents with a migrant background reported the fear of becoming infected more strongly, without the share of these respondents dominating the specified occupational group or any other. This can possibly be explained by varying levels of informational awareness and education. The fact that this plays an important role in the spread of infections is proven by numerous studies on other infectious diseases [51,52,53]. 

With respect to age, there was a slight tendency for fears to decrease with increasing age, including the fear of infecting others, deciding on treatment or concern for patient safety due to increasing workload. An increase in age also led to a decrease in alcohol consumption and, at the same time, an increase in how protection by employers or authorities was rated. This finding indicates that age could be seen as a protective factor against anxiety and stress. One could intuitively attribute this to more experience and training, but it may also be explained as an underestimation of the risk of infection with COVID-19 as age increases [54]. Data from the general population in the COVID-19 pandemic also show decreasing prevalence rates for stress, anxiety and depression from younger to older people [55].

Respondents that came into contact with contaminated materials/infected individuals reported higher scores across nearly all COVID-19-associated problem areas than respondents who had no such contact. This has already been shown in similar studies [56]. Accordingly, it appears critical that frontline healthcare workers should be given priority in relief and support services when treating COVID-19 patients. The ability to work from home also increased perceived protection by employers and authorities. However, for respondents with a migrant background this was true in only a few instances. This finding could be related to lower levels of trust in the state and/or employers in addition to poorer education, and points to the need to take long-term, sustainable measures to provide employees from other cultural backgrounds with the same level of security as people without a migrant background. Here, too, circumstances that require an untested and new way of working appear to lead to higher stress. To better prepare for emergency situations in the future, the ability to work partly or completely from home should be evaluated and used on a more regular basis. 

The balance between work and family life is a factor that has long been underestimated in terms of the perceived impact on employees [57,58]. Our research also reveals that respondents who have caregiving responsibilities in their family in addition to their healthcare jobs experienced higher levels of stress from the increased workload and change in tasks. It seems important in this context that employers should offer such employees special support or that, from a political point of view, opportunities should be created in which caregiving responsibilities can be met without negatively impacting workloads. This finding is consistent with the results of a qualitative study on intensive care nurses by Feeley et al., who identified the “home-work interface” as an extra-organizational factor with a significant influence on the perception of occupational stress and its psychological impact on the well-being of respondents [59]. The current life situation of the individual also seems significant: respondents who lived alone reported either equal or increased stress as well as higher use of dysfunctional coping strategies. Based on this, one could conclude that society and a sense of community can be seen as a protective factor and loneliness and isolation as a risk factor [60]. The importance of social support as a resilience factor among healthcare workers in the pandemic is also demonstrated by the work of Schmuck et al., 2021 [61] and Morganstein et al., 2021 [62]. Given the persistent and repetitive measures in addition to the dynamics of the pandemic since the first wave, this fact seems all the more critical. Fiorillo and Gorwood formulate very general implications on this aspect: the importance of communication with close people, the importance of social networks, professional support services and, finally, psychiatric counseling and treatment services. The latter address an important task for mental health professionals in the health sector. They should be actively involved in the planning and organization of healthcare in the COVID-19 pandemic, in addition to when it comes to supporting colleagues from other disciplines [63]. Kuzman et al. (2020) recommend education, guidance and the development of administrative frameworks, as well as the production of documents with relevant information. They should, according to the authors, be advocates for people with mental disorders and illnesses, anticipate their needs and plan protective measures in the pandemic [64]. Stewart and Applebaum also propose that mental health professionals should be involved in the development of self-help, group or individual support as well as treatment for distressed colleagues and their families. In addition, mental health professionals should also participate in COVID-19 decision-making boards to uphold the rights of colleagues undergoing mental distress. They can participate in education for health professionals, the public and policy makers to inform them about mental distress. Leaders in health professions, especially the psychiatric specialties, should alert policy makers and authorities to the long-term consequences of mentally distressed health professionals and the need for targeted support services tailored to health professionals [65]. 

### Limitations

The results presented here are from a cross-sectional survey and do not allow any causal inferences to be drawn about the development of stress and protective factors. Further measurement points are planned, so that multicentric longitudinal analyses can be carried out in the long term. Due to the lack of a validated questionnaire to assess the specific burden of healthcare workers during this pandemic, we adapted a questionnaire developed by Matsuishi et al. [31] during the H1N1 pandemic. However, our questionnaire achieved a comparable and reasonable internal consistency. The planned repeated survey will allow us to additionally assess the retest reliability. Furthermore, it should be noted that the majority of respondents were women and this very unequal sample size prevailed in other conditions as well. Hence, these results are not representative of the entire population of healthcare workers, and any disparities presented here must be considered with this in mind. It remains unclear whether the participants were so impacted that they agreed to the survey or whether it was those who were the most impacted who tended to avoid participation due to personal hardship and lack of time.

## 5. Conclusions

The illustrated results of the survey identify the potential factors of influence on increased stress due to the COVID-19 pandemic and protective factors for German-speaking healthcare workers in a large sample for the first time. Based on these results, the COVID-19 pandemic has greatly impacted the following groups, work environments and living situations: women, employees with a migrant background, younger employees, individuals with private obligations to care for children and dependents, men with regard to dysfunctional coping strategies, people living alone and, when compared to other occupational groups, frontline workers, such as nurses/paramedics and medical technicians. This leads to different areas of action in which employers can take preventative measures by offering the following support: establishing a departmental representative for women as a point of contact, offering internal psychosocial counseling services to assist with leisure activities and developing functional coping strategies to deal with stress, the establishment of virtual peer groups for discussion and reflection on individual stress factors, the introduction of company-provided opportunities for recreational activities, coordinating defined recreational teams where people who live alone have the option to pursue joint activities in adherence with pandemic-related constraints, virtual recreational meetings or courses offered by the employer, educational and information brochures in different languages, contact persons for people with a migrant background and virtual information events. Flexible working hours with additional personnel and “substitutes” on standby appear to be especially important. For younger workers in particular, regular supervision groups led by experienced colleagues and additional training opportunities should be provided. Because of the special role played by nurses/paramedics and medical technicians during pandemics, they should receive preventive training as part of their education and career development, and they should be members within a task force responsible for pandemics.

## Figures and Tables

**Table 1:** Distribution of respondents to different occupational groups.

Occupation		Physicians N = 1906	Nurses and Paramedics N = 1422	Medical Technicians N = 1727	Therapist/Educator Occupations N = 1601	Other N = 886	Total
Gender	Male	744 (41.87%)	379 (21.33%)	221 (12.44%)	210 (11.82%)	223 (12.55%)	1777 (23.6%)
	Female	1157 (20.13%)	1036 (18.03%)	1506 (26.21%)	1388 (24.15%)	660 (11.48%)	5747 (76.2%)
	Diverse	5 (27.78%)	7 (38.88%)	0 (0.00%)	3 (16.67%)	3 (16.67%)	18 (0.2%)
Age groups	18–30	154 (10.92%)	441 (31.28%)	377 (26.74%)	239 (16.95%)	199 (14.11%)	1410 (18.7%)
	31–40	453 (26.58%)	342 (20.07%)	389 (22.83%)	305 (17.90%)	215 (12.62%)	1704 (22.6%)
	41–50	466 (26.80%)	290 (16.68%)	391 (22.48%)	410 (23.58%)	182 (10.47%)	1739 (23.1%)
	51–60	604 (28.04%)	296 (13.74%)	489 (22.70%)	519 (24.09%)	246 (11.42%)	2154 (28.6%)
	61–70	208 (41.27%)	50 (9.92%)	81 (16.07%)	124 (24.60%)	41 (8.13%)	504 (6.7%)
	>70	21 (67.74%)	3 (9.68%)	0 (0.00%)	4 (12.90%)	3 (9.68%)	31 (0.4%)
Contaminated materials/infected individuals	Contact	956 (26.55%)	866 (24.05%)	1285 (35.68%)	252 (7.00%)	242 (6.72%)	3601 (47.7%)
	No contact	950 (24.11%)	556 (14.11%)	442 (11.22%)	1349 (34.23%)	644 (16.34%)	3941 (52.3%)
Home office	Yes	467 (28.32%)	84 (5.09%)	166 (10.07%)	609 (36.93%)	323 (19.59%)	1649 (21.9%)
	No	1439 (24.42%)	1338 (22.70%)	1561 (26.49%)	992 (16.83%)	563 (9.55%)	5893 (78.1%)
Caregiving	Yes	1363 (28.91%)	770 (16.33%)	969 (20.56%)	1083 (22.97%)	529 (11.22%)	4714 (62.5%)
	No	543 (19.20%)	652 (23.06%)	758 (26.80%)	518 (18.32%)	357 (12.62%)	2828 (37.5%)
Migrant background	Yes	238 (28.88%)	166 (20.15%)	166 (20.15%)	132 (16.02%)	122 (14.81%)	824 (10.9%)
	No	1667 (24.83%)	1254 (18.68%)	1561 (23.25%)	1468 (21.86%)	764 (11.38%)	6714 (89.1%)
Living situation	Living alone	303 (18.39%)	380 (23.06%)	427 (25.91%)	311 (18.87%)	227 (13.77%)	1648 (21.9%)
	Not living alone	1603 (27.20%)	1042 (17.68%)	1300 (22.06%)	1290 (21.89%)	659 (11.18%)	5894 (78.1%)
Place of work	Clinic	1039 (21.56%)	1228 (25.49%)	1296 (26.90%)	506 (10.50%)	749 (15.55%)	4818 (63.9%)
	Doctor’s office	482 (67.70%)	4 (0.56%)	173 (24.30%)	48 (6.74%)	5 (0.70%)	712 (9.4%)
	Community health center	46 (19.83%)	11 (4.74%)	160 (68.97%)	5 (2.16%)	10 (4.31%)	232 (3.1%)
	Other	321 (18.30%)	177 (10.09%)	93 (5.30%)	1042 (59.41%)	121 (6.90%)	1754 (23.3%)

**Table 2:** Problems and protective factors associated with COVID-19.

During the First Wave of the Pandemic…	N	M	SD
**Problems**			
… I was afraid of becoming infected.	7542	2.66	1.24
… I was afraid of infecting loved ones or family.	7542	3.27	1.34
… I was stressed by the increased workload.	7542	2.52	1.24
… I was stressed by changes to my tasks.	7542	2.70	1.31
… I was reluctant to go to work.	7542	1.91	1.10
… I had problems sleeping.	7542	2.53	1.36
… I felt physically or mentally exhausted.	7542	3.06	1.30
… I was afraid of having to choose who would receive care and who would not.	7542	1.53	0.98
... I was worried my patients would die without seeing their relatives again.	7542	2.24	1.44
... I felt that patient safety suffered from the higher workload.	7542	2.10	1.19
… I smoked more cigarettes.	7542	1.40	1.02
… I drank more alcohol.	7542	1.62	1.10
… I took more antidepressants/sedatives.	7542	1.14	0.59
**Protective Factors**			
… I felt protected by the actions of national and local authorities.	7542	3.10	1.07
… I felt protected as an employee by measures taken by my employer.	7542	3.25	1.17

**Table 3:** Distribution of response options on COVID-19-associated problems and protective factors (reported as n (%)).

	1 = Strongly Disagree	2 = Somewhat Disagree	3 = Partly Agree, Partly Disagree	4 = Somewhat Agree	5 = Strongly Agree
Fear of infection	1500 (19.9%)	2271 (30.1%)	1820 (24.1%)	1217 (16.1%)	734 (9.7%)
Infecting others	956 (12.7%)	1488 (19.7%)	1332 (17.7%)	2062 (27.3%)	1704 (22.6%)
Workload	1904 (25.2%)	2219 (29.4%)	1593 (21.1%)	1245 (16.5%)	581 (7.7%)
Change in tasks	1766 (23.4%)	1885 (25.0%)	1484 (19.7%)	1636 (21.6%)	771 (10.2%)
Reluctant to go to work	3659 (48.5%)	1978 (26.2%)	1069 (14.2%)	604 (8.0%)	232 (3.1%)
Sleep problems	2441 (32.4%)	1526 (20.2%)	1497 (19.8%)	1326 (17.6%)	752 (10.0%)
Physical or mental exhaustion	1237 (16.4%)	1349 (17.9%)	1747 (23.2%)	2148 (28.5%)	1061 (14.1%)
Patient care decisions	5348 (70.9%)	1119 (14.8%)	541 (7.2%)	366 (4.9%)	168 (2.2%)
Patient dies without seeing relatives again	3626 (48.1%)	1142 (15.1%)	923 (12.2%)	1008 (13.4%)	843 (11.2%)
Patient safety	3131 (41.5%)	2015 (26.7%)	1245 (16.5%)	795 (10.5%)	356 (4.7%)
Nicotine consumption	6370 (84.5%)	260 (3.4%)	264 (3.5%)	371 (4.9%)	277 (3.7%)
Alcohol consumption	5310 (70.4%)	727 (9.6%)	726 (9.6%)	582 (7.7%)	197 (2.6%)
Antidepressant use	7035 (93.3%)	190 (2.5%)	150 (2.0%)	104 (1.4%)	63 (0.8%)
Protection by authorities	690 (9.1%)	1395 (18.5%)	2453 (32.5%)	2474 (32.8%)	530 (7.0%)
Protection by employer	685 (9.1%)	1339 (17.8%)	2017 (26.7%)	2403 (31.9%)	1098 (14.6%)

**Table 4:** Analysis of variance on different COVID-19-associated problems and protective factors.

Problems and Protective Factors Associated with COVID-19	Testing Statistics of the Analysis of Variance
Fear of infection	F(4, 7537) = 25.73, *p* < 0.001, η² = 0.01
Infecting others	F(4, 7537) = 51.78, *p* < 0.001, η² = 0.03
Workload	F(4, 7537) = 9.98, *p* < 0.001, η² = 0.01
Change in tasks	F(4, 7537) = 35.16, *p* < 0.001, η² = 0.02
Reluctant to go to work	F(4, 7537) = 28.59, *p* < 0.001, η² = 0.02
Sleep problems	F(4, 7537) = 16.22, *p* < 0.001, η² = 0.01
Physical or mental exhaustion	F(4, 7537) = 14.72, *p* < 0.001, η² = 0.01
Patient care decisions	F(4, 7537) = 62.91, *p* < 0.001, η² = 0.03
Patient dies without saying goodbye	F(4, 7537) = 101.79, *p* < 0.001, η² = 0.05
Patient safety	F(4, 7537) = 66.63, *p* < 0.001, η² = 0.03
Nicotine consumption	F(4, 7537) = 65.19, *p* < 0.001, η² = 0.03
Alcohol consumption	F(4, 7537) = 9.44, *p* < 0.001, η² = 0.01
Antidepressant use	F(4, 7537) = 8.50, *p* < 0.001, η² = 0.01
Protection by authorities	F(4, 7537) = 43.62, *p* < 0.001, η² = 0.02
Protection by employer	F(4, 7537) = 23.98, *p* < 0.001, η² = 0.01

**Table 5:** Means and standard deviations for selected COVID-19-associated problem areas and related protective factors across different age groups.

	Infecting Others	Patient Dies without Seeing Relatives again	Patient Safety	Alcohol Consumption	Protection by Authorities	Protection by Employer
Test statistics	F(5, 7561) = 36.90, *p* < 0.001, η² = 0.02	F(5, 7561) = 5.12, *p* < 0.001, η² = 0.003	F(5, 7561) = 18.79, *p* < 0.001, η² = 0.01	F(5, 7561) = 10.18, *p* < 0.001, η² = 0.01	F(5, 7561) = 8.41, *p* < 0.001, η² = 0.01	F(5, 7561) = 5.66, *p* < 0.001, η² = 0.004
18–30 (N = 1411)	3.59 (1.29)	2.40 (1.46)	2.33 (1.27)	1.71 (1.17)	2.96 (1.04)	3.13 (1.12)
31–40 (N = 1706)	3.43 (1.31)	2.27 (1.45)	2.16 (1.22)	1.73 (1.18)	3.07 (1.06)	3.21 (1.15)
41–50 (N = 1745)	3.20 (1.35)	2.15 (1.43)	2.05 (1.16)	1.62 (1.09)	3.15 (1.05)	3.29 (1.17)
51–60 (N = 2161)	3.08 (1.35)	2.21 (1.43)	1.98 (1.14)	1.53 (1.00)	3.17 (1.10)	3.31 (1.21)
61–70 (N = 507)	2.99 (1.33)	2.23 (1.49)	1.96 (1.09)	1.46 (0.92)	3.14 (1.14)	3.31 (1.27)
>70 (N = 32)	2.53 (1.48)	2.16 (1.54)	1.72 (1.02)	1.50 (1.19)	2.97 (1.33)	2.88 (1.56)

Differences in means presented were significant across age groups at a level of *p* < 0.001.

## Data Availability

There is no consent from the participants to pass on the data. With justified reason, the data can be requested via the corresponding author.
